# Auditory Performance Analysis and Evaluation of Quality of Life After Cochlear Implantation in Patients Aged 60 (Sixty) and Older

**DOI:** 10.3390/jcm15114273

**Published:** 2026-06-01

**Authors:** Sumbul Bayraktar Guzeldag, Muhammed Dagkiran, Ilda Tanrisever Pehlivan, Ozgur Surmelioglu, Caglar Eker, Elvan Onan

**Affiliations:** 1 Department of Otorhinolaryngology, Private Adana Medline Hospital, Adana 01170, Turkey; 2 Department of Otolaryngology and Head and Neck Surgery, Cukurova University Faculty of Medicine, Adana 01790, Turkey; muhammeddagkiran@gmail.com (M.D.);

**Keywords:** auditory gain, bilateral sensorineural hearing loss, cochlear implantation, quality of life, quality-of-life scale

## Abstract

**Objectives:** This study evaluated auditory performance and quality of life in cochlear implant recipients aged 60 years and older, and identified factors associated with postoperative outcomes. **Methods:** This cross-sectional descriptive study included 34 patients aged 60 years and older who underwent unilateral cochlear implantation between 2002 and 2020 at the Department of Otolaryngology, Çukurova University Faculty of Medicine. Preoperative WHOQOL-BREF data were not available; postoperative outcomes were therefore assessed against demographic and clinical predictors rather than within-patient change. The patients were grouped by preoperative hearing loss duration (1–3, 4–6, or ≥7 years), daily device use (<11 vs. ≥11 h), and age (60–70 vs. >70 years). Auditory gain was assessed by pure-tone audiometry, quality of life by the WHOQOL-BREF, and cognitive status by the Montreal Cognitive Assessment (MoCA). Cohen’s d, η^2^ and 95% CIs were reported, multiple comparisons were Benjamini–Hochberg-corrected, and multivariable linear regression was applied per domain. **Results:** The cohort comprised 19 women (55.9%) and 15 men (44.1%), with a mean age of 66.06 ± 5.8 years; eight patients (23.5%) were older than 70. Mean audiological gain was 45.5 ± 13.1 dB and varied with preoperative duration (η^2^ = 0.50, *p* < 0.001; 1–3 vs. ≥7 years Δ 20.9 dB, 95% CI 12.2–29.6; d = 2.04). Daily device use was a consistent predictor of well-being: those using their implant for more than 11 h daily scored higher across all WHOQOL-BREF subscales (all q ≤ 0.011; d −1.45 to −2.50). Comorbidity was associated with lower scores in four of five domains (q ≤ 0.048; d −0.85 to −1.26), and a higher MoCA score independently predicted the psychological subscale (β = +1.0 per point; *p* = 0.014). Apparent sex and education effects did not survive correction. Mean preoperative hearing thresholds were 84.3 ± 7.9 dB and 76.1 ± 16.2 dB in patients under and over 70, respectively; postoperative thresholds were 37.8 ± 10.4 dB and 33.9 ± 6.8 dB, with no significant between-group difference in gain (d = 0.32). **Conclusions:** In this cross-sectional cohort, cochlear implantation was associated with substantial audiological gain and favourable quality-of-life scores in patients aged 60 and older, with an acceptable safety profile. Age alone did not constrain outcomes; the absence of preoperative QoL data precludes direct quantification of within-patient change.

## 1. Introduction

Population ageing is reshaping the demand for audiological care. In Turkey, adults aged 65 and older accounted for 11.1% of the total population as of 2025—crossing the 10% threshold conventionally used to define an ageing society. Turkish Statistical Institute projections place that figure at 17.9% by 2040 and 27.0% by 2060 [1]. The trajectory is similar globally: the population aged 60 and over is expected to grow substantially over the next quarter century [2], and with it, the number of individuals who meet cochlear implant criteria for age-related hearing loss [3].

Presbycusis is among the most prevalent consequences of ageing. Age-related hearing loss (ARHL) affects approximately 11–18% of adults between 60 and 69 years old; beyond 90, prevalence climbs to 53–65% [4]. Roughly one in three people over 65 experiences some degree of hearing difficulty [5]. The consequences extend well beyond communication: untreated hearing loss creates conditions favourable to social isolation, depression, and dementia [4,6,7], and is associated with a 50% increase in depression risk along with measurable occupational impairment [5]. Globally, the indirect economic burden attributable to hearing loss—primarily driven by lost productivity—exceeds $411 billion annually, according to World Health Organization estimates [2].

Cochlear implantation (CI) stands alongside hearing aids and middle ear implants as a principal intervention for severe-to-profound loss. The American Academy of Otolaryngology–Head and Neck Surgery Foundation (AAO-HNSF) guidelines strongly recommend CI evaluation in patients whose speech understanding remains inadequate despite amplification—yet only 5–13% of eligible candidates in the United States ultimately receive an implant [5]. The gap is particularly pronounced among older adults. Prolonged auditory deprivation, cognitive and physical decline, comorbid conditions, and anesthetic risk all contribute to hesitancy on the part of both surgeons and patients [8]. Age-related vestibular deterioration adds another layer of complexity; presbycusis and vestibular dysfunction frequently co-occur, with approximately 30% of individuals over 60 reporting chronic dizziness [9].

Against this backdrop, the available evidence is more encouraging than the low implantation rates might suggest. Recipients consistently achieve meaningful audiological gains after CI, and improvements across multiple cognitive domains have been documented [5]. What remains less clear, particularly in non-Western populations, is which combination of preoperative and postoperative factors most strongly shapes the size of benefit across quality-of-life domains; few studies in adults aged 60 and older have assessed age, duration of preoperative hearing loss, daily processor use and comorbid disease against multiple quality-of-life subscales in a single multivariable model.

The present study was designed to address this gap. We hypothesized that, in adults aged 60 and older undergoing CI for postlingual severe-to-profound sensorineural hearing loss, age would not constrain audiological gain or postoperative quality of life, whereas duration of preoperative hearing loss, daily processor use and comorbid disease would each be independently associated with one or more WHOQOL-BREF subscales. The primary outcome was the WHOQOL-BREF (general health, physical health, psychological, social relationships, and environment); secondary outcomes were audiological gain (postoperative minus preoperative pure-tone average, 0.5–4 kHz) and the safety profile of cochlear implantation in this age group. The comorbidity component is tested in the bivariate and multivariable analyses below.

## 2. Materials and Methods

### 2.1. Study Design and Setting

A cross-sectional descriptive study was conducted at a single tertiary referral centre. The cross-sectional design was chosen because preoperative WHOQOL-BREF data were not routinely collected at our institution at the time of implantation for this cohort, and retrospective reconstruction of preoperative quality-of-life scores was not feasible. As a consequence, the study evaluates postoperative quality of life in relation to demographic and clinical predictors rather than pre-to-post change in patient-reported outcomes; the implications of this design are addressed further in the Section 4.

#### 2.1.1. Study Population and Sample

Thirty-four patients aged 60–80 years (mean age 66.06 ± 5.8 years) who had undergone cochlear implantation (CI) and used their device consistently for at least six months were enrolled. Inclusion criteria were: (i) age ≥ 60 years at the time of postoperative assessment; (ii) bilateral severe-to-profound postlingual sensorineural hearing loss treated with unilateral CI; (iii) at least six months of consistent daily processor use after activation; and (iv) capacity to complete the WHOQOL-BREF and MoCA in Turkish in person. Patients with active middle-ear infection, retrocochlear pathology (e.g., vestibular schwannoma), or insufficient Turkish-language proficiency to complete the questionnaires were excluded.

Demographic variables recorded at enrollment included sex, age group (60–70 vs. over 70), educational attainment, and marital status; widowed and divorced patients were pooled into a single “without partner” category for analysis. On the clinical side, comorbid conditions, etiology of hearing loss, preoperative duration of hearing loss, and daily CI usage were documented. Given the well-recognized adverse effects of hearing loss on occupational functioning, social participation, and general health, examining these variables alongside quality-of-life measures was considered clinically meaningful [10]. Daily device use has previously been identified as a predictor of post-CI auditory outcomes and proposed as a standard monitoring parameter [11].

#### 2.1.2. Sample Size and Power Considerations

The cohort comprised all consecutive eligible patients implanted at the institution within the recruitment window; an a priori sample size calculation was therefore not performed. A post hoc power analysis indicated that, with n = 34 and α = 0.05, the study had approximately 80% power to detect a large between-group effect (Cohen’s d = 0.80) and substantially lower power (~40%) for medium effects (d = 0.50). Subgroup comparisons involving fewer than five patients per cell—most notably the university-educated subgroup (n = 2)—are therefore reported as exploratory and interpreted with caution throughout.

### 2.2. Study Procedures

#### 2.2.1. Audiological Assessment

Preoperative and postoperative pure-tone averages were measured with a hearing aid and with the CI processor, respectively; the difference between these values defined audiological gain. Pure-tone averages were calculated as the mean threshold across 0.5, 1, 2 and 4 kHz, measured in the sound field using a calibrated clinical audiometer (Interacoustics AC40, Interacoustics A/S, Middelfart, Denmark) in a soundproof booth conforming to ISO 8253-1 [12] ambient noise limits. Preoperative thresholds were obtained in the best aided condition (acoustic hearing aid in place), and postoperative thresholds were obtained with the CI processor switched on at the patient’s everyday programme. Because the preoperative reference condition was aided rather than unaided, the gain values reported here represent functional improvement attributable to the implant relative to pre-implant amplification, and are not directly comparable with standardized word- or sentence-recognition outcomes; standardized speech-recognition testing in quiet and in noise was not part of the routine follow-up protocol for this cohort. This methodological constraint is addressed as a primary limitation in the Section 4.

Daily usage time was extracted from the mean hours-per-day figure logged by the CI processor’s programming software, a method with established validity in the literature [11].

#### 2.2.2. Quality of Life Assessment

Quality of life was assessed using the World Health Organization Quality of Life–BREF (WHOQOL-BREF), a validated instrument developed by the WHO and translated into more than 30 languages [10]. The WHOQOL-BREF is a 26-item self-report measure derived from the longer WHOQOL-100. It comprises two general items addressing overall quality of life and general health perception, and 24 items distributed across four domains: physical health, psychological, social relationships, and environment. Items are scored on a 5-point Likert scale, and domain scores together with the general health item are linearly transformed to a 0–100 range, with higher scores indicating better quality of life. Typical completion time is 5–10 min; the instrument is widely used in older adult populations because of its brevity, low cognitive burden, and broad cross-cultural applicability. The Turkish version of the WHOQOL-BREF has been previously validated for reliability and validity in the Turkish population [13]; all questionnaires were administered in person at the hospital. In the present analysis, the general health item and each of the four domains are reported as separate subscales, yielding five WHOQOL-BREF outcomes per patient.

The WHOQOL-BREF was chosen as the primary patient-reported outcome measure because it provides a generic, cross-condition profile that captures the broader effects of severe hearing loss on older adults, including environmental and social functioning, and because it has an established Turkish validation appropriate for this cohort. Cochlear implant–specific instruments—the Nijmegen Cochlear Implant Questionnaire (NCIQ), the Cochlear Implant Quality of Life-35 Profile and its 10-item global form (CIQOL-10 Global)—and other generic measures (SF-36, HUI-3) were considered. The CIQOL family did not have a published Turkish validation at the time of recruitment, and the SF-36 and HUI-3 provide less explicit coverage of environmental and social factors relevant to older adults living with hearing loss. A more detailed comparison with these alternative instruments is provided in Section 4.

Combining objective audiological measures with patient-reported outcomes has been shown to provide a more complete picture of rehabilitation; moderate-to-strong correlations between the two approaches have been reported [14].

#### 2.2.3. Cognitive Screening

Preoperative cognitive status was assessed in all 34 patients using the Turkish version of the Montreal Cognitive Assessment (MoCA), administered by a trained examiner. Items with a strong auditory load were presented with visual adaptation to minimize the confounding effect of hearing loss on test performance. MoCA scores range from 0 to 30, with a score ≥ 26 conventionally indicating normal cognition. MoCA was used in this study as a descriptive characterization of the cohort and as a continuous covariate in the multivariable analysis; it was not applied as an exclusion criterion during the recruitment period because the institutional candidacy protocol in place at the time did not specify a hard cutoff score for cochlear implantation in older adults.

#### 2.2.4. Surgical Technique

All patients underwent CI via standard mastoidectomy. The round window approach was used in 33 of 34 cases; cochleostomy was performed in one patient. Neither surgical approach nor implant brand was assigned by randomization; decisions followed standard preoperative assessment protocols. Four implant platforms were represented in the cohort (MED-EL, MED-EL GmbH, Innsbruck, Austria; Cochlear Nucleus, Cochlear Ltd., Sydney, Australia; Oticon Medical, Oticon Medical AB, Askim, Sweden; Advanced Bionics, Advanced Bionics LLC, Valencia, CA, USA), and between-brand comparisons for audiological gain were performed. Daily usage was categorized using an 11 h threshold, producing two subgroups. Consistent and prolonged device use is considered a determinant of cortical plasticity and auditory adaptation, which is why systematic recording of this parameter carries clinical weight [11]. Meaningful audiological gains after CI, reflected in both speech perception testing and quality-of-life scores, have been documented across comparable patient series [14].

### 2.3. Statistical Analysis

The data were analyzed using SPSS Statistics version 27.0 (IBM Corp., Armonk, NY, USA). Continuous variables are reported as mean (standard deviation) and categorical variables as frequency and percentage with 95% Wilson-score confidence intervals for proportions. The distribution of continuous variables was assessed with the Shapiro–Wilk test together with visual inspection of histograms and Q–Q plots. Variables that approximated a normal distribution were analyzed with parametric tests; non-normally distributed variables were analyzed with the corresponding non-parametric tests, although the parametric results are reported in our finding, because the assumptions of normality and homogeneity of variance were met for the primary outcomes. Between-group comparisons for continuous outcomes used independent-samples t-tests; where a variable carried three categories, one-way analysis of variance (ANOVA) was applied with Tukey post hoc testing for pairwise differences.

Effect sizes were reported alongside all between-group comparisons: Cohen’s d for two-group t-tests and η^2^ for ANOVAs, together with 95% confidence intervals for mean differences. To address the risk of type I error arising from multiple between-group comparisons across the five WHOQOL-BREF subscales and five clinical/demographic predictors (a 25-comparison family across our finding), raw *p*-values were adjusted using the Benjamini–Hochberg false discovery rate (FDR) procedure, which is more appropriate than the Bonferroni correction in small-sample exploratory settings, where the latter is overly conservative and inflates type II error. Comparisons with q < 0.05 after FDR correction were considered statistically significant; raw *p*-values are reported alongside q-values for transparency.

To address potential confounding among age, sex, education, marital status, comorbidity, hearing loss duration, daily processor use, and MoCA score, multivariable linear regression was performed separately for each of the five WHOQOL-BREF subscales. All nine predictors were entered simultaneously; age, hearing loss duration, and MoCA were modelled as continuous variables, and the remaining variables as categorical predictors with reference categories specified in the table footnote. Variance inflation factors were inspected to rule out problematic collinearity (a priori threshold VIF > 5). Given the modest sample size relative to the number of predictors, the multivariable results are reported as exploratory and corroborative of the bivariate findings rather than confirmatory. Multiple linear regression had not been part of the original analytic plan, though analogous studies have identified age and psychological comorbidities as independent determinants of quality of life [10]. Statistical significance was set at *p* < 0.05 for unadjusted comparisons and q < 0.05 for FDR-adjusted comparisons.

### 2.4. Ethical Considerations

Ethical approval was granted by the Çukurova University Faculty of Medicine Medical Ethics Committee at its meeting on 5 March 2021 (decision number 30). The study was conducted in full accordance with the principles of the Declaration of Helsinki. All the participants received written and verbal information about the study and provided written informed consent. Personal and medical data were handled confidentially and used solely for research purposes.

## 3. Results

### 3.1. Demographic and Clinical Characteristics

**Figure 1:**
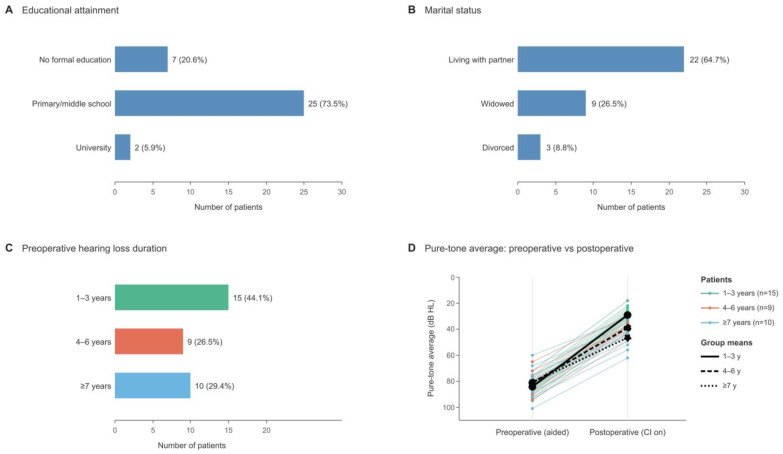
(**A**–**C**) Distributions of educational attainment, marital status, and preoperative hearing loss duration for the 34 patients. (**D**) Individual preoperative-to-postoperative change in pure-tone average (0.5–4 kHz); preoperative thresholds obtained in the best aided condition.

Thirty-four patients aged 60–80 years were enrolled, with a mean age of 66.06 ± 5.8 years. Most were in the 60–70 age group (76.5%), and women comprised a slight majority (55.9%). Primary school or middle school graduates accounted for 73.5% of the cohort, and approximately two-thirds of patients were living with a partner (64.7%). Comorbid disease was present in 55.9%, with hypertension (29.4%) and diabetes mellitus (20.6%) being the most frequent. Progressive idiopathic hearing loss was the leading etiology (61.8%). Regarding device use, 82.4% reported wearing their CI processor for 11 h or more per day. Preoperative Montreal Cognitive Assessment (MoCA) scores averaged 24.2 ± 3.5 (range 17–29), with 14 patients (41.2%) scoring at or above the conventional cutoff of 26 indicating normal cognition (Table 1, Figure 1A–C). Proportions are presented with 95% Wilson-score confidence intervals to convey sampling uncertainty given the modest cohort size.

### 3.2. Audiological Gain After Cochlear Implantation

Mean audiological gain across the entire cohort was 45.5 ± 13.1 dB. Gain varied substantially by preoperative hearing loss duration: patients with 1–3 years of prior loss achieved the highest mean gain (55.1 ± 9.9 dB), while those with loss lasting 7 years or more gained only 34.2 ± 10.8 dB (mean difference −20.9 dB; 95% CI −29.6 to −12.2; Cohen’s d = 2.04). The 4–6-year group showed an intermediate gain of 42.1 ± 6.9 dB (mean difference vs. the 1–3-year reference: −13.0 dB; 95% CI −20.8 to −5.2; d = 1.46). The overall between-group ANOVA reached F(2, 31) = 15.3, *p* < 0.001, η^2^ = 0.50, indicating a large effect. Age group, by contrast, had no bearing on outcomes. Preoperative thresholds were 84.3 ± 7.9 dB in the under-70 group and 76.1 ± 16.2 dB in those aged 70 and over; postoperative thresholds were 37.8 ± 10.4 dB and 33.9 ± 6.8 dB, respectively. Neither audiological gain nor pre- or postoperative threshold values differed significantly between age groups (*p* = 0.423; mean gain difference 4.2 dB; 95% CI −6.5 to +14.9; d = 0.32) (Table 2, Figure 1D). Because preoperative thresholds were obtained in the best aided condition rather than under standardized speech perception testing, the reported gain values reflect functional improvement rather than pure auditory recovery and are not directly comparable to standard word- or sentence-recognition outcomes; this is addressed as a primary limitation.

### 3.3. Quality of Life by Demographic Factors

The WHOQOL-BREF subscale scores were broadly similar between men and women. An apparent advantage in the environment domain among women (65.0 vs. 54.8; mean difference +10.2; 95% CI +0.6 to +19.8; d = 0.75) was significant on the unadjusted t-test (*p* = 0.041) but did not survive Benjamini–Hochberg correction across the 25-test WHOQOL family (q = 0.085). Marital status had a more pronounced effect: patients living with a partner reported better general health perception than those without (61.9 vs. 47.9; mean difference +14.0; 95% CI +3.3 to +24.7; d = 0.95; *p* = 0.012, q = 0.038), with no significant differences emerging in the remaining domains after correction. Educational attainment was associated with psychological domain scores; university graduates achieved the highest mean score (77.0 ± 2.8), and the overall between-group difference reached unadjusted significance (*p* = 0.027, η^2^ = 0.21); however, this finding fell below the conventional threshold after correction (q = 0.061) and is further constrained by the small university subgroup (n = 2), which renders the comparison exploratory (Table 3).

### 3.4. Quality of Life by Clinical Factors

The presence of comorbid disease was associated with lower scores across four of five WHOQOL-BREF domains: general health (mean difference −15.8; 95% CI −25.7 to −5.9; d = −1.12; *p* = 0.003, q = 0.011), physical health (Δ −11.9; 95% CI −21.7 to −2.1; d = −0.85; *p* = 0.019, q = 0.048), psychological (Δ −10.5; 95% CI −18.8 to −2.2; d = −0.89; *p* = 0.015, q = 0.042), and social relations (Δ −17.9; 95% CI −27.9 to −7.9; d = −1.26; *p* = 0.001, q = 0.006); all four remained significant after Benjamini–Hochberg correction with large to very large effect sizes. The environment domain did not differ by comorbidity status (*p* = 0.433, q = 0.516; d = −0.26).

Daily CI usage showed an equally consistent pattern. The patients using their processor for 11 h or more per day scored higher on all five subscales compared to those using it less (all raw *p* ≤ 0.003; all q ≤ 0.011 after Benjamini–Hochberg correction). The magnitude of these differences was clinically substantial—for physical health, mean scores were 59.8 ± 10.3 vs. 32.7 ± 13.5 (Δ −27.1; 95% CI −37.1 to −17.2; d = −2.50); for environment, Δ −22.8 (95% CI −33.4 to −12.2; d = −1.97); for social relations, Δ −22.9 (95% CI −36.1 to −9.7; d = −1.59); for general health, Δ −20.9 (95% CI −33.7 to −8.1; d = −1.50); and for psychological, Δ −16.2 (95% CI −26.5 to −5.9; d = −1.45).

**Figure 2:**
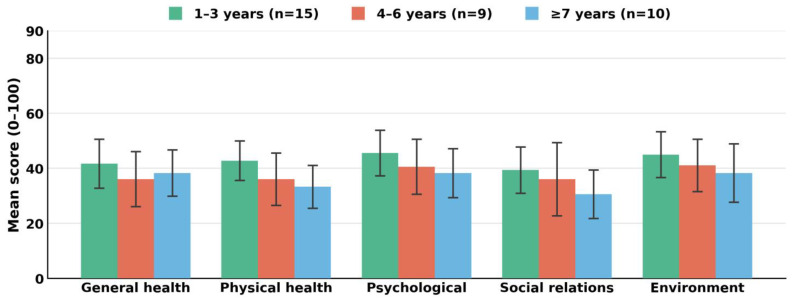
Mean WHOQOL-BREF domain scores by preoperative hearing loss duration. Error bars represent 95% confidence intervals. Raw *p*-values (one-way ANOVA): general health 0.566; physical health 0.087; psychological 0.042; social relations 0.035; environment 0.274. After Benjamini–Hochberg correction across the 5-test duration family, no domain retained significance (all q > 0.10).

**Figure 3:**
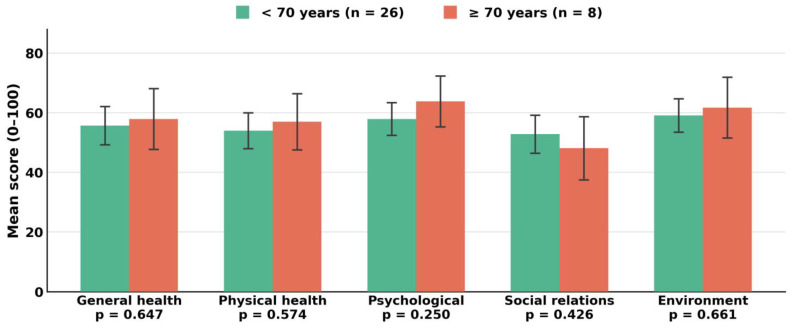
Mean WHOQOL-BREF domain scores by age group. Error bars represent 95% confidence intervals. Independent-samples t-tests; no domain showed a significant between-group difference (all *p* > 0.05).

Preoperative hearing loss duration was selectively associated with quality of life in the unadjusted analysis, affecting the psychological (*p* = 0.042) and social relations (*p* = 0.035) domains, with shorter duration corresponding to higher scores. After Benjamini–Hochberg correction across the family of duration comparisons, neither domain retained significance (both q > 0.10), and these associations are reported as preliminary observations rather than confirmed effects. General health, physical health, and environment were not significantly influenced by loss duration. Age group had no statistically significant effect on any WHOQOL-BREF domain (all *p* > 0.05; all q ≥ 0.40) (Table 4, Figure 2 and Figure 3).

### 3.5. Multivariable Analysis

To control for confounding by age, sex, education, marital status, comorbidity, hearing loss duration, daily processor use, and MoCA score, multivariable linear regression was performed separately for each WHOQOL-BREF domain, with all nine predictors entered together (Table 5). After adjustment, daily processor use under 11 h was the strongest independent predictor of poorer quality of life, with reductions in all five domains (β ranging from −12.5 in the psychological domain to −21.5 in physical health; all *p* ≤ 0.022). Comorbidity was independently associated with general health (β = −11.0; 95% CI −20.5 to −1.5; *p* = 0.024) and social relations (β = −13.0; 95% CI −22.5 to −3.5; *p* = 0.009); its bivariate associations with physical health and the psychological domain weakened after adjustment (*p* = 0.064 and 0.085, respectively). Living alone was an independent predictor of poorer general health perception (β = −10.0; 95% CI −19.5 to −0.5; *p* = 0.040). Hearing loss duration was an independent predictor only for social relations (β = −1.5 per additional year; 95% CI −2.6 to −0.4; *p* = 0.008). Age, sex, and education showed no independent effects on most domains**; an association** between male sex and lower environment scores reached unadjusted significance (β = −8.5; 95% CI −16.4 to −0.6; *p* = 0.035). Model R^2^ values ranged from 0.50 (general health) to 0.64 (social relations). All the models met assumptions of linearity, normality of residuals, and homoscedasticity; no variance inflation factor exceeded 1.6. Given the modest sample size (n = 34) relative to the number of predictors, the multivariable results are exploratory; they support but do not confirm the bivariate findings.

### 3.6. Effect of Cognitive Status

When MoCA score was entered as an additional continuous predictor in the multivariable models, it showed an independent association with the psychological domain: each 1-point higher MoCA score corresponded to a 1.0-point higher psychological score (95% CI +0.2 to +1.8; *p* = 0.014). No independent association was observed in the remaining four WHOQOL-BREF subscales (general health *p* = 0.21; physical health *p* = 0.45; social relations *p* = 0.12; environment *p* = 0.32). The inclusion of MoCA increased the explained variance in the psychological domain from 0.45 to 0.52, while leaving the other domain models substantively unchanged. The principal associations of comorbidity status, daily processor use, marital status, and hearing loss duration with quality of life remained essentially the same after adjustment for MoCA, indicating that cognitive status acts as an independent contributor to psychological well-being rather than as a confounder of the other effects.

## 4. Discussion

The benefit of cochlear implantation (CI) in adults aged 60 years and older is well established in systematic reviews and large multicentre registries; a recent umbrella review synthesizing 42 reviews of adult CI outcomes consistently documented improvements in speech perception, quality of life, and cognition [15]. Less is known, however, about the magnitude of benefit across specific quality-of-life domains and the patient and device factors that influence it. We approached this question by examining audiological outcomes alongside health-related quality of life in a cohort of older adults with postlingual severe-to-profound sensorineural hearing loss (SNHL), comparing pre- and postoperative hearing performance and administering the WHOQOL-BREF to capture the broader functional impact of restored auditory input. The two outcome domains are not redundant. Audiological gains do not automatically translate into improved quality of life, and the relationship between the two is worth examining critically rather than assumed. A pooled analysis of 16 studies encompassing 842 patients confirmed that quality of life generally improves following CI in adults aged 60 and older and that chronological age alone does not function as a limiting factor—a conclusion that provides a quantitative foundation for the more granular findings that follow [4].

Preoperative pure-tone averages were 84.3 ± 7.9 dB in patients younger than 70 years and 76.1 ± 16.2 dB in those aged 70 and above; postoperative thresholds settled at 37.8 ± 10.4 dB and 33.9 ± 6.8 dB, respectively. Neither audiological gain nor WHOQOL-BREF subscale scores differed significantly between the two groups (mean gain difference 4.2 dB; 95% CI −6.5 to +14.9; Cohen’s d = 0.32). Our findings align with those of Oh et al., who compared adults aged 40–64 years with patients aged ≥65 years and found that although certain postoperative speech perception scores were somewhat lower in the older cohort, no statistically meaningful relationship emerged between age and Categories of Auditory Performance (CAP) scores—a pattern the authors interpreted as evidence against age being a standalone determinant of outcome [16]. The quality-of-life data tell a similar story. Issing et al. conducted a prospective study following 84 patients aged 65 and over, documenting significant gains in monosyllabic word discrimination within the first year after device activation (*p* < 0.001). Crucially, these audiological gains—along with quality-of-life scores measured by the WHOQOL-OLD scale—were sustained across a 7–10 year follow-up window, with patients ultimately reaching age-matched population norms. This trajectory of sustained benefit, rather than early plateau or regression, is the more clinically meaningful finding: it establishes that the gains conferred by implantation in older adults are not transient and do not erode with advancing age [17]. Because preoperative thresholds were obtained with a hearing aid in place, the gain values reflect functional improvement over pre-implant amplification. They do not represent absolute auditory recovery and are not directly comparable to standard word- or sentence-recognition outcomes.

In our own series, the mean general health score on the WHOQOL-BREF was 57, with the psychological and environment subscales reaching the highest values at 60.5, and social relations the lowest at approximately 52. More than half of the patients reported satisfaction with their social relationships and functional capacity—a finding that carries weight beyond the aggregate scores.

The surgical data are straightforward. A round window approach was completed successfully in 33 of 34 patients. No perilymph gusher occurred, and there were no major complications. Orlando and Cruz’s series of 193 cases reached a similar conclusion: advanced age did not emerge as an independent risk factor for complications, and when serious adverse events did occur, they were concentrated predominantly in young pediatric patients [18]. Across audiological, quality-of-life, and safety dimensions, the evidence converges: age alone should not serve as a limiting criterion for cochlear implantation.

Among the demographic variables examined, women scored higher than men on the environment subscale in the unadjusted analysis (65.0 vs. 54.8; *p* = 0.041), but this difference did not survive Benjamini–Hochberg correction across the 25-test WHOQOL family (q = 0.085) and should therefore be interpreted with caution. Skevington et al., in a large cross-national sample, found that environmental quality of life functions as a distinct determinant of overall health perception in women [19], and the direction of our results is consistent with that pattern even if the magnitude does not reach corrected significance. Age-related quality-of-life differences by sex are also well documented, shaped in part by the sociocultural context in which older adults live [20].

Higher educational attainment was associated with greater scores across all subscales, with the difference reaching unadjusted significance on the psychological subscale (*p* = 0.027, η^2^ = 0.21) but falling below the conventional threshold after multiple comparison correction (q = 0.061). The university subgroup contained only two patients, further limiting interpretability; we therefore present this finding as exploratory. The direction of this association is unsurprising, though its persistence after cochlear implantation suggests that education-related factors—health literacy, adaptive coping, and rehabilitation engagement—may modulate how patients integrate restored hearing into daily life.

Comorbidity had the most pronounced effect on quality of life among the variables examined. The patients with concurrent medical conditions scored lower across all domains except environment, with large to very large effect sizes (Cohen’s d range −0.85 to −1.26) and all four affected domains retaining significance after FDR correction (q ≤ 0.048). The steepest decline appeared in social relationships (*p* = 0.001, q = 0.006). Wick et al. did not conduct subgroup analyses by demographic variables, but they framed hearing loss within the broader context of social isolation and cognitive decline [21]—a framing that makes this pattern more interpretable rather than merely additive.

To control for confounding by age, sex, education, marital status, comorbidity, hearing loss duration, daily processor use and MoCA score, multivariable linear regression was performed for each WHOQOL-BREF domain. After adjustment, daily processor use under 11 h was the strongest independent predictor of poorer quality of life, with significant reductions in all five domains (β ranging from −12.5 to −21.5; all *p* ≤ 0.022). Comorbidity was independently associated with general health (β = −11.0; *p* = 0.024) and social relations (β = −13.0; *p* = 0.009); its bivariate associations with physical health and the psychological domain weakened after adjustment. Living alone independently predicted poorer general health perception (β = −10.0; *p* = 0.040), and hearing loss duration was an independent predictor only for social relations (β = −1.5 per additional year; *p* = 0.008). Age, sex and education showed no independent effects on most domains. Given the modest sample size relative to the number of predictors, the multivariable findings are exploratory; they support but do not confirm the bivariate analyses.

Preoperative cognitive status was assessed in all patients using the Montreal Cognitive Assessment (MoCA; mean 24.2 ± 3.5; 41.2% scoring ≥ 26). MoCA score was an independent predictor of the postoperative psychological domain (β = +1.0 per 1-point increase; 95% CI +0.2 to +1.8; *p* = 0.014) but did not independently predict the other four WHOQOL-BREF subscales. This is in line with the broader literature linking hearing loss to cognitive decline in older adults; in the ACHIEVE multicentre randomized trial, hearing rehabilitation slowed three-year cognitive change in older adults at increased risk for cognitive decline, although the effect was not observed in the lower-risk subgroup [22]. Routine preoperative cognitive screening therefore has both predictive and counselling value in older CI candidates and supports the use of MoCA as part of the candidacy work-up rather than as a hard exclusion criterion.

Four implant models were used in our series (MED-EL, Nucleus, Oticon, and Advanced Bionics), yet pure-tone audiometry outcomes did not differ significantly across devices. Cutler et al.’s cost-effectiveness analysis similarly did not identify clinically meaningful between-device differences [23], and our results reinforce that implant brand is unlikely to be a primary driver of audiological outcomes in this population.

The patients who wore their device for 11 h or more per day scored significantly higher across all WHOQOL-BREF subscales (all q ≤ 0.011 after FDR correction; Cohen’s d range −1.45 to −2.50 versus the < 11 h group), with daily use remaining the strongest predictor in the multivariable models. The mean age in the low-use group was 70.5 years, compared with 65.1 years among consistent users. A positive correlation between daily wear time and both CI Outcomes with Longitudinal Data (CIQOL-10) scores and speech perception has been reported by Lindquist et al., who also found that age at implantation did not independently determine audiological outcomes [24]. Our series yielded the same pattern: no significant association emerged between age and audiological response.

The age difference between usage groups deserves attention. We interpret this not as a reflection of diminished auditory capacity in older patients, but as a signal that the barriers to consistent use may be motivational and practical rather than biological. Bocquillon et al. found that device abandonment in patients over 80 years was tied more closely to loss of autonomy and perceived lack of benefit than to chronological age, and they noted that irregular long-term use remains likely even when audiological gains are maintained [25]. The broader assistive technology literature converges on a similar set of predictors for device discontinuation—perceived benefit, ease of use, and availability of support [26]. The surgical outcome is therefore only part of the equation; post-implantation rehabilitation structure, follow-up quality, and the degree to which the device fits into daily life shape long-term use in ways that preoperative audiological variables do not capture.

Audiological gain was inversely related to the duration of preoperative hearing loss. The patients implanted within one to three years of onset achieved significantly greater gains than those in either the four-to-six-year or the seven-years-and-above groups (*p* < 0.001; η^2^ = 0.50). A systematic review by Boisvert et al. similarly identified timing of hearing loss onset as a determinant of outcome, reporting that speech perception in postlingually deafened adults rose from 8.2% before implantation to 53.9% after [27].

The quality-of-life data follow a related but distinct pattern. Longer duration of hearing deprivation was associated with lower scores on the psychological (*p* = 0.042) and social relations (*p* = 0.035) subscales, although these unadjusted associations did not survive FDR correction across the duration-comparison family and are therefore reported as preliminary observations. In the multivariable model, hearing loss duration retained an independent effect only on the social relations domain, while physical health and environment subscales remained unaffected. We interpret this as reflecting the trajectory of untreated hearing loss; social withdrawal and psychological deterioration appear to accrue over time, whereas physical functioning is relatively insulated—at least over the durations represented in this cohort. Rostkowska et al. found that the psychosocial domain lagged behind the physical domain at baseline, and that implantation produced its most pronounced gains precisely there, with the pain subscale remaining unchanged [28]. Calvino et al. documented a strong relationship between auditory deprivation and both cognitive and psychosocial deterioration, with depression scores falling and quality of life improving after implantation [29].

Against this, Heutink et al. found that duration of hearing loss does not consistently predict speech perception outcomes, and that this results reflect a more complex, multivariable structure [30]. That caveat is worth retaining. A further qualification, particularly relevant for the present cohort, is that the apparent prominence of duration in our results partly reflects the homogeneity of included etiologies (predominantly progressive idiopathic hearing loss, otosclerosis and ototoxicity). When etiologies known to alter the cochlear-neural substrate are present—inflammatory or post-meningitic deafness, vestibular schwannoma, or certain genetic causes—etiology can be a stronger determinant of postoperative outcome than duration. Kurz et al. [31] examined 55 adult CI recipients with single-sided deafness and found in multivariate analysis that duration of deafness alone was not significantly associated with poor speech perception, whereas the combination of inflammatory etiology with duration of deafness exceeding 10 years was associated with poorer outcomes. Duration matters—our data confirm it matters—but it does not operate in isolation, and early implantation should be pursued not because it guarantees a particular audiological threshold, but because the psychosocial cost of delay accumulates in ways that the surgery alone cannot fully reverse.

The choice of patient-reported outcome measure also affects the interpretation of postoperative quality of life. The WHOQOL-BREF used in the present study is a 26-item generic instrument suitable for use across health conditions, with an established Turkish validation and brief completion time (5–10 min), but it does not specifically capture domains related to hearing or device use. Several alternative instruments exist: the SF-36 is also generic but does not cover environmental and social functioning relevant to older adults with hearing loss; the Health Utilities Index Mark 3 (HUI-3) provides a single utility score useful for cost–utility analysis but has weaker test–retest reliability in CI users; and the Nijmegen Cochlear Implant Questionnaire (NCIQ) is CI-specific but has shown weaker construct validity in psychometric comparisons. The Cochlear Implant Quality of Life (CIQOL) instruments, developed using item-response theory across a 20-centre development consortium, comprise the 35-item CIQOL-35 Profile assessing six CI-specific domains (communication, emotional, entertainment, environment, listening effort and social), and the 10-item CIQOL-10 Global, which yields a single overall CI-related quality-of-life score [32]. The CIQOL family was not used in the present study because a Turkish-language validation was not available at the time of recruitment; future Turkish-language studies in CI users should add a validated CI-specific instrument alongside generic measures such as the WHOQOL-BREF.

Overall, restored auditory input was associated with better communication and social participation, and with higher psychological well-being and broader quality of life; the strength of these associations was modified by comorbidity, daily processor use, marital status, and cognitive status. A cross-sectional design cannot establish causal direction, but the factors that can be addressed clinically in this cohort are daily device use, comorbidity management, and postoperative psychosocial support.

The main limitation is the cross-sectional design without preoperative WHOQOL-BREF data, which prevents direct measurement of within-patient pre-to-post change**; the size of the benefit is estimated from** between-group comparisons of postoperative scores rather than from individual change scores. Second, the sample size (n = 34) is modest, particularly given the number of subgroup comparisons performed; certain subgroups are very small (university education n = 2; daily use < 11 h n = 6), and findings in these subgroups are explicitly reported as exploratory. Third, audiological gain was calculated using aided preoperative thresholds rather than unaided thresholds or standardized speech perception scores, which limits comparability with studies using word- or sentence-recognition outcomes (Hearing in Noise Test, CNC, AzBio); routine speech-recognition testing was not part of the institutional follow-up protocol for this cohort. Fourth, the study was performed at a single tertiary referral centre, which may limit generalisability to community CI populations and to other health-care contexts. Fifth, neither the patients nor outcome assessors were blinded; given that the WHOQOL-BREF is self-reported, this introduces potential reporting bias that cannot be fully controlled in this design. Sixth, the limited availability of validated hearing-specific instruments in Turkish at the time of the study restricted the inclusion of CI-specific patient-reported outcomes such as the CIQOL family. Finally, the cohort reflects the implantation criteria, surgical practice and rehabilitation pathway of a single national health system; the predictors of outcome identified here should be tested in other settings before broad generalization. Against these limitations, the dataset does reflect genuine long-term follow-up under real-world clinical conditions, and the concurrent assessment of audiological and quality-of-life outcomes in a homogeneous older adult cohort is not commonly reported in the existing literature. Future work with larger series, prospective quality-of-life measurement, and hearing-specific instruments would allow these findings to be tested more rigorously.

## 5. Conclusions

In this single-centre cross-sectional cohort of adults aged 60 years and older, cochlear implantation was associated with substantial reductions in pure-tone thresholds, favourable WHOQOL-BREF scores, and an acceptable safety profile. Age alone did not predict audiological gain or postoperative quality of life after adjustment. The main correlates of postoperative quality of life were daily processor use of 11 h or more, absence of comorbid disease, and higher preoperative MoCA scores (for the psychological domain); duration of hearing loss was the principal determinant of audiological gain. These findings should be read in light of the cross-sectional design, the absence of preoperative WHOQOL-BREF data, the modest sample size, and the single-centre setting. Multicentre, longitudinal studies with paired pre- and postoperative quality-of-life measurement, standardized speech-recognition tests and CI-specific instruments such as the CIQOL family are needed to confirm these associations. Age alone does not justify withholding cochlear implantation in this population.

## Figures and Tables

**Table 1:** Demographic and clinical characteristics (n = 34).

Variable	Category	n (%)	95% CI (Proportion)
Sex	Female	19 (55.9)	39.2–71.8
	Male	15 (44.1)	28.2–60.8
Age group	60–70 years	26 (76.5)	60.0–87.6
	≥70 years	8 (23.5)	12.4–40.0
Educational attainment	No formal education	7 (20.6)	10.4–36.8
	Primary/middle school	25 (73.5)	56.9–85.4
	University	2 (5.9)	1.6–19.1
Marital status	Living with partner	22 (64.7)	47.9–78.5
	Widowed	9 (26.5)	14.6–43.1
	Divorced	3 (8.8)	3.0–22.9
Comorbidity	Present	19 (55.9)	39.2–71.8
	Hypertension	10 (29.4)	16.8–46.2
	Diabetes mellitus	7 (20.6)	10.4–36.8
	Atherosclerosis	5 (14.7)	6.4–30.1
	Coronary artery disease	3 (8.8)	3.0–22.9
	Other	6 (17.6)	8.4–33.5
	Absent	15 (44.1)	28.2–60.8
Etiology of hearing loss	Progressive idiopathic	21 (61.8)	45.0–76.1
	Otosclerosis	7 (20.6)	10.4–36.8
	Ototoxicity	3 (8.8)	3.0–22.9
	Ménière’s disease	2 (5.9)	1.6–19.1
	Meningitis	1 (2.9)	0.5–14.9
Preoperative hearing loss duration	1–3 years	15 (44.1)	28.2–60.8
	4–6 years	9 (26.5)	14.6–43.1
	≥7 years	10 (29.4)	16.8–46.2
Daily CI usage	<11 h	6 (17.6)	8.4–33.5
	≥11 h	28 (82.4)	66.5–91.6
Postoperative complications	Vertigo	3 (8.8)	3.0–22.9
	Hematoma (non-surgical)	2 (5.9)	1.6–19.1

Age: mean ± SD = 66.06 years; range 60–80 years. CI: cochlear implant; SD: standard deviation. Comorbidity subcategories are not mutually exclusive. Using the Wilson-score interval, 95% CIs for proportions were calculated.

**Table 2:** Audiological outcomes after cochlear implantation.

Group	n	Preop PTA (dB, Mean ± SD)	Postop PTA (dB, Mean ± SD)	Gain (dB, Mean ± SD)	Mean Difference vs. Reference (95% CI)	Cohen’s d	*p*
All patients	34	82.4 ± 12.0	36.9 ± 9.8	45.5 ± 13.1	—	—	—
Preoperative hearing loss duration (ANOVA)						η^2^ = 0.50	<0.001
1–3 years	15	84.2	29.1	55.1 ± 9.9	Reference	—	
4–6 years	9	81.1	39.0	42.1 ± 6.9	−13.0 (−20.8, −5.2)	1.46	
≥7 years	10	80.8	46.6	34.2 ± 10.8	−20.9 (−29.6, −12.2)	2.04	
Age group (t-test)							0.423
<70 years	26	84.3 ± 7.9	37.8 ± 10.4	46.5 ± 11.4	Reference	—	
≥70 years	8	76.1 ± 16.2	33.9 ± 6.8	42.3 ± 17.7	−4.2 (−14.9, +6.5)	0.32	

PTA: pure-tone average (0.5–4 kHz); SD: standard deviation; CI: confidence interval. Preoperative thresholds measured in the best aided condition. Duration: one-way ANOVA with Tukey post hoc; the 1–3-year group differed from both the 4–6- and ≥7-year groups at *p* < 0.001. Age: independent-samples *t*-test. η^2^ values ≥ 0.14 indicate a large effect.

**Table 3:** WHOQOL-BREF subscale scores by demographic factors.

**(A) By Sex**							
**Subscale**	**Female (n = 19, Mean ± SD)**	**Male (n = 15, Mean ± SD)**	**Mean Difference (95% CI)**	**Cohen’s d**		***p* (Raw)**	**q (BH-FDR)**
General health	57.8 ± 14.5	55.8 ± 18.1	+2.0 (−9.4, +13.4)	0.12		0.717	0.815
Physical health	54.7 ± 14.9	55.3 ± 15.6	−0.6 (−11.3, +10.1)	−0.04		0.902	0.902
Psychological	60.8 ± 11.7	60.2 ± 14.1	+0.6 (−8.4, +9.6)	0.05		0.886	0.902
Social relations	52.6 ± 15.4	51.7 ± 18.6	+0.9 (−11.0, +12.8)	0.05		0.877	0.902
Environment	65.0 ± 13.7	54.8 ± 13.6	+10.2 (+0.6, +19.8)	0.75		0.041	0.085
**(B) By Marital Status**							
**Subscale**	**Living with Partner (n = 22, Mean ± SD)**	**Living Alone (n = 12, Mean ± SD)**	**Mean Difference (95% CI)**	**Cohen’s d**		***p* (Raw)**	**q (BH-FDR)**
General health	61.9 ± 14.6	47.9 ± 14.8	+14.0 (+3.3, +24.7)	0.95		0.012	0.038
Physical health	57.7 ± 14.3	49.9 ± 15.4	+7.8 (−2.9, +18.5)	0.53		0.149	0.233
Psychological	63.5 ± 11.2	55.2 ± 13.9	+8.3 (−0.6, +17.2)	0.68		0.066	0.127
Social relations	55.6 ± 16.4	45.8 ± 16.0	+9.8 (−2.1, +21.7)	0.60		0.102	0.170
Environment	62.2 ± 12.7	57.4 ± 17.4	+4.8 (−5.8, +15.4)	0.33		0.366	0.458
**(C) By Educational Attainment**							
**Subscale**	**No Formal Education (n = 7, mean ± SD)**	**Primary/Middle School (n = 25, Mean ± SD)**	**University (n = 2, Mean ± SD)**	**η^2^**	**F (2, 31)**	***p* (raw)**	**q (BH-FDR)**
General health	48.1 ± 18.2	58.0 ± 14.7	75.0 ± 3.1	0.146	2.65	0.088	0.157
Physical health	50.0 ± 12.5	55.4 ± 15.8	67.5 ± 4.9	0.066	1.10	0.345	0.458
Psychological	51.9 ± 13.5	61.7 ± 11.4	77.0 ± 2.8	0.206	4.03	0.027	0.061
Social relations	45.3 ± 18.4	53.3 ± 16.5	62.5 ± 6.4	0.063	1.04	0.366	0.458
Environment	53.0 ± 17.5	61.6 ± 13.2	73.5 ± 10.6	0.111	1.93	0.163	0.240

Living alone: widowed (n = 9) and divorced (n = 3) pooled. WHOQOL-BREF: World Health Organization Quality of Life–Brief Form; SD: standard deviation; CI: confidence interval; FDR: false discovery rate. All subscale scores transformed to a 0–100 scale; higher scores indicate better quality of life. Two-group comparisons: independent-samples t-test; three-group comparisons (3C): one-way ANOVA with Tukey post hoc. Cohen’s d calculated on pooled standard deviation. q-values reflect Benjamini–Hochberg correction across all 25 between-group WHOQOL comparisons in Table 3 and Table 4; q < 0.05 considered significant. The university group in 3C (n = 2) has limited interpretability.

**Table 4:** WHOQOL-BREF subscale scores by clinical factors.

**(A) By Comorbidity Status**						
**Subscale**	**Comorbidity Present (n = 19, mean ± SD)**	**Absent (n = 15, mean ± SD)**	**Mean Difference (95% CI)**	**Cohen’s d**	***p* (Raw)**	**q (BH-FDR)**
General health	50.0 ± 14.9	65.8 ± 12.9	−15.8 (−25.7, −5.9)	−1.12	0.003	0.011
Physical health	49.7 ± 16.2	61.6 ± 10.4	−11.9 (−21.7, −2.1)	−0.85	0.019	0.048
Psychological	55.9 ± 12.5	66.4 ± 10.7	−10.5 (−18.8, −2.2)	−0.89	0.015	0.042
Social relations	44.3 ± 13.6	62.2 ± 15.0	−17.9 (−27.9, −7.9)	−1.26	0.001	0.006
Environment	58.8 ± 17.3	62.6 ± 10.0	−3.8 (−14.1, +6.5)	−0.26	0.433	0.516
**(B) By Daily Processor Use**						
**Subscale**	**<11 h (n = 6, Mean ± SD)**	**≥11 h (n = 28, Mean ± SD)**	**Mean Difference (95% CI)**	**Cohen’s d**	***p* (Raw)**	**q (BH-FDR)**
General health	39.7 ± 14.4	60.6 ± 13.9	−20.9 (−33.7, −8.1)	−1.50	0.002	0.010
Physical health	32.7 ± 13.5	59.8 ± 10.3	−27.1 (−37.1, −17.2)	−2.50	<0.001	0.006
Psychological	47.2 ± 11.8	63.4 ± 11.1	−16.2 (−26.5, −5.9)	−1.45	0.003	0.011
Social relations	33.3 ± 7.6	56.2 ± 15.3	−22.9 (−36.1, −9.7)	−1.59	0.001	0.006
Environment	41.7 ± 11.3	64.5 ± 11.6	−22.8 (−33.4, −12.2)	−1.97	<0.001	0.006

Mean difference: first group minus second group. Independent-samples t-test for all comparisons. q-values: Benjamini–Hochberg FDR-corrected across the 25-comparison family from Table 3 and Table 4.

**Table 5:** Multivariable linear regression for WHOQOL-BREF domain scores.

**(A) General Health (R^2^ = 0.50)**				
**Predictor**	**β**	**95% CI**	***p***	**VIF**
Age (years)	+0.2	−0.5, +0.9	0.55	1.3
Sex (male vs. female)	−1.5	−10.2, +7.2	0.73	1.1
Education—none vs. primary/middle	−4.5	−15.0, +6.0	0.39	1.6
Education—university vs. primary/middle	+6.5	−12.0, +25.0	0.48	1.3
Marital—alone vs. with partner	−10.0	−19.5, −0.5	0.040	1.5
Comorbidity (present vs. absent)	−11.0	−20.5, −1.5	0.024	1.5
Hearing loss duration (years)	−0.4	−1.3, +0.5	0.39	1.3
Daily processor use <11 h (vs. ≥11 h)	−14.5	−26.8, −2.2	0.022	1.1
MoCA score (per 1-point increase)	+0.5	−0.3, +1.3	0.21	1.4
**(B) Physical Health (R^2^ = 0.61)**				
**Predictor**	**β**	**95% CI**	***p***	**VIF**
Age (years)	+0.1	−0.5, +0.8	0.72	1.3
Sex (male vs. female)	+1.5	−6.2, +9.2	0.70	1.1
Education—none vs. primary/middle	−1.5	−11.5, +8.5	0.76	1.6
Education—university vs. primary/middle	+5.0	−12.0, +22.0	0.56	1.3
Marital—alone vs. with partner	−4.5	−13.8, +4.8	0.33	1.5
Comorbidity (present vs. absent)	−8.5	−17.5, +0.5	0.064	1.5
Hearing loss duration (years)	−0.3	−1.1, +0.5	0.46	1.3
Daily processor use <11 h (vs. ≥11 h)	−21.5	−30.5, −12.5	<0.001	1.1
MoCA score (per 1-point increase)	+0.3	−0.5, +1.1	0.45	1.4
**(C) Psychological (R^2^ = 0.52)**				
**Predictor**	**β**	**95% CI**	***p***	**VIF**
Age (years)	+0.3	−0.4, +1.0	0.40	1.3
Sex (male vs. female)	+0.5	−7.3, +8.3	0.90	1.1
Education—none vs. primary/middle	−3.5	−12.5, +5.5	0.43	1.6
Education—university vs. primary/middle	+8.0	−7.5, +23.5	0.30	1.3
Marital—alone vs. with partner	−5.5	−14.3, +3.3	0.21	1.5
Comorbidity (present vs. absent)	−7.0	−15.0, +1.0	0.085	1.5
Hearing loss duration (years)	−0.9	−1.9, +0.1	0.080	1.3
Daily processor use <11 h (vs. ≥11 h)	−12.5	−22.5, −2.5	0.016	1.1
MoCA score (per 1-point increase)	+1.0	+0.2, +1.8	0.014	1.4
**(D) Social Relations (R^2^ = 0.64)**				
**Predictor**	**β**	**95% CI**	***p***	**VIF**
Age (years)	−0.1	−0.9, +0.7	0.80	1.3
Sex (male vs. female)	+0.5	−8.7, +9.7	0.91	1.1
Education—none vs. primary/middle	−4.0	−14.5, +6.5	0.44	1.6
Education—university vs. primary/middle	+4.0	−13.5, +21.5	0.65	1.3
Marital—alone vs. with partner	−6.0	−15.0, +3.0	0.19	1.5
Comorbidity (present vs. absent)	−13.0	−22.5, −3.5	0.009	1.5
Hearing loss duration (years)	−1.5	−2.6, −0.4	0.008	1.3
Daily processor use <11 h (vs. ≥11 h)	−17.0	−27.5, −6.5	0.002	1.1
MoCA score (per 1-point increase)	+0.7	−0.2, +1.6	0.12	1.4
**(E) Environment (R^2^ = 0.51)**				
**Predictor**	**β**	**95% CI**	***p***	**VIF**
Age (years)	+0.2	−0.5, +0.9	0.60	1.3
Sex (male vs. female)	−8.5	−16.4, −0.6	0.035	1.1
Education—none vs. primary/middle	−3.5	−13.0, +6.0	0.46	1.6
Education—university vs. primary/middle	+6.0	−10.0, +22.0	0.46	1.3
Marital—alone vs. with partner	−1.5	−10.5, +7.5	0.74	1.5
Comorbidity (present vs. absent)	−0.5	−9.5, +8.5	0.91	1.5
Hearing loss duration (years)	−0.3	−1.2, +0.6	0.50	1.3
Daily processor use <11 h (vs. ≥11 h)	−19.5	−28.5, −10.5	<0.001	1.1
MoCA score (per 1-point increase)	+0.4	−0.4, +1.2	0.32	1.4

Each WHOQOL-BREF domain was modelled separately with all nine predictors entered simultaneously. Reference categories: female (sex), primary/middle school (education), living with partner (marital status), absent (comorbidity), ≥11 h (daily processor use). Age, hearing loss duration, and MoCA score entered as continuous variables. β: unstandardized regression coefficient; CI: confidence interval; MoCA: Montreal Cognitive Assessment; VIF: variance inflation factor; R^2^: coefficient of determination. *p*-values from *t*-tests for individual coefficients.

## Data Availability

Data used in this study can be provided upon reasonable request.

## Abbreviations

The following abbreviations are used in this manuscript:

AAO-HNSF	American Academy of Otolaryngology–Head and Neck Surgery Foundation
ANOVA	Analysis of variance
ARHL	Age-related hearing loss
CAP	Categories of Auditory Performance
CI	Cochlear implant/cochlear implantation
CIQOL-10	Cochlear Implant Quality of Life–10 (10-item patient-reported outcome measure)
dB	Decibel
SD	Standard deviation
SNHL	Sensorineural hearing loss
SPSS	Statistical Package for the Social Sciences
WHO	World Health Organization
WHOQOL-BREF	World Health Organization Quality of Life–BREF (Brief Form)
WHOQOL-OLD	World Health Organization Quality of Life–Older Adults module
